# Using the modified Delphi technique to develop a framework for interprofessional education during international electives in health professions training institutions in Sub-Saharan Africa

**DOI:** 10.3389/fmed.2023.1225475

**Published:** 2023-10-18

**Authors:** Faith Nawagi, Ian Guyton Munabi, Andre Vyt, Sarah Kiguli, Tracy Rabin, Firdouza Waggie, Aloysius Gonzaga Mubuuke

**Affiliations:** ^1^School of Medicine, Makerere University College of Health Sciences, Kampala, Uganda; ^2^School of Biomedical Sciences, Makerere University College of Health Sciences, Kampala, Uganda; ^3^Faculty of Medicine and Health Sciences, Ghent University, Ghent, Belgium; ^4^School of Medicine, Yale University, New Haven, CT, United States; ^5^Faculty of Community and Health Sciences, University of the Western Cape, Bellville, South Africa

**Keywords:** framework, interprofessional education, interprofessional collaboration, international electives, Africa

## Abstract

**Background:**

International electives provide a learning platform where interprofessional education and collaborative practice (IPECP) skills can be cultivated. However, hardly any frameworks to guide the implementation of interprofessional education (IPE) during international electives, especially in the context of low-income settings have been published. To address this gap, this study used the modified Delphi approach to develop an IPE framework guide for international electives to be used by health professions training institutions in Sub-Saharan Africa.

**Methods:**

A rapid literature review and a study among students and faculty in four African health professional training institutions were done to inform the process. This was followed by the modified Delphi technique that used three Delphi rounds with a panel of eight experts to build consensus on the final framework for IPE during international electives. The level of consensus was set at ≥70% on each of the statements in all rounds.

**Results:**

Out of the 52 statements in round 1 (*n* = 37, 71%) reached consensus while (*n* = 15, 29%) of the statements did not reach consensus and were discussed in round 2. Round 2 led to 42 statements to be utilized for round 3. In round 3, all statements (42) reached a consensus and an IPE framework to guide the implementation of international electives was developed. The framework consists of three sections. Section one highlights the various IPECP competencies to be gained by learners in the areas of teamwork, interprofessional communication, roles and responsibilities of interprofessional collaborative practice, values and ethics of interprofessional collaboration, and reflection and evaluation of oneself and the team. Section two gives guidance on the structuring of the IPE international electives in health professional training institutions. This includes subsections on operational/institutional needs, acculturation considerations, teaching strategies, assessment strategies, mode of delivery, and public health considerations. Section three consists of the various practical guides and approaches that health professional training institutions could use according to what works best in their setting.

**Conclusion:**

The modified Delphi technique was an adequate approach to aid the development of an IPE framework to guide implementation during international electives in various health professional training institutions.

## Introduction

Interprofessional education (IPE) in health is defined as when two or more professions learn with, from, and about each other to enable effective collaboration and improve health outcomes (1). A few institutions in Africa have made strides in the integration of IPE competencies in the curriculum through didactic and experiential learning, especially during community-based field attachment modules and within the training of the basic sciences (2). However, efforts to offer IPE and build frameworks that include self-evaluation to guide interprofessional education and collaborative practice (IPECP) implementation and learning in international electives, especially in Africa remain minimal, yet key (3).

The World Health Organization has supported IPE by developing a framework for action on IPECP that proposed new models of educating healthcare providers (1). This framework further proposed syncing education with health care systems with an interprofessional approach to lead to enhanced quality of health care (1). Despite its relevance till today, it did not provide learning environment-specific guidelines to implement IPE among learners. Specifically, it did not provide guidelines on teaching, assessment, and orientation approaches for IPE in various learning environments like the clinical, community, simulation, lectures, and international electives, among others.

The Interprofessional Education Collaborative (IPEC) defined the key competencies of IPE as; values/ethics for IPECP, roles/responsibilities for IPECP, interprofessional communication practices, interprofessional teamwork, and team-based practice (4). All these competencies should be implemented with approaches that are family-centered, patient-oriented, and for the community at large (4). These approaches have a similarity with those of the Canadian Interprofessional Health Collaborative (CIHC) (5). In addition, the European Interprofessional Practice and Education Network (EIPEN) has developed a framework for interprofessional collaboration in health care which involves key competencies that include: consult and collaborate, plan and manage, handle issues and opportunities, refer and transfer, and reflect and evaluate in all areas of work, all involving an interprofessional approach (6).

Despite the relevance of all the above frameworks on IPE competencies that also include aspects of respect for culture and uniqueness of other professions, it is challenging to develop guidelines for implementing IPE in various learning contexts including international electives. International electives are defined as the time of learning where students have a choice of where to learn, and the discipline they should be taking across borders from their own country (7). These mainly occur in another country at a particular host institution that has a partnership agreement that may be unilateral, bilateral, or multilateral with the student’s home institution (8). International electives as a form of teaching and learning are part of various health professions training institutions globally (9, 10). The majority of the international electives are clinical or community-based and often have the elective visiting students interact with local students and faculty of the same discipline at the host institution (11). The majority of the students are from the medicine and nursing disciplines, respectively, among others (11), with hardly any interaction or joint activities (12). International electives are often 4 weeks at the undergraduate level and often students have to find sponsorships or self-fund (12). Most of the evaluation is done through reflective reports, scoring sheets from the home institution of the student which are given to the supervising faculty, and post-elective surveys (12). International electives have been documented to enhance the learners’ global perspectives, knowledge and skills, interpersonal and professional development, and positive attitudes to better health service delivery (13). Furthermore, international electives provide a learning platform where IPECP skills can be cultivated especially if offered with a structured approach (3).

IPE international electives though scarce, have been implemented between Vanderbilt University USA and Nicaragua Eye Hospital and were perceived to be effective to enable students gain IPECP (14). However, hardly any frameworks to guide the implementation of IPE during international electives, especially in the context of low-income settings, where social care issues and aspects of resource limitation can determine interprofessional health care to a large extent have been published (15). Health professional line of command and practice, which is an aspect of culture, largely differ in various African countries and globally (16). Some cadres of professionals may be able to perform some tasks during clinical care while in other countries it may not be possible (16). Given that IPE international electives require mobility to another country, a guide for intercultural orientation hardly exists.

Much of the research on IPE and learning has been informed by psychological and cognitive theories pushing aside the role of the learning environment and cultural context. This study was guided by the social constructivism theory, a social-cultural theory advanced by Vygotsky et al. (17). This theory postulates that individuals work together to construct and develop ideas through dialogue that builds on prior knowledge and understanding. Here, the importance of culture, context, and social interaction are key to shaping one’s knowledge gain. For the social constructivist, knowledge is socially and culturally constructed (18). Individuals create meaning through their interactions with each other and with the environment they live in. In relation to structuring learning in IPE international electives, learners within an international elective placement do interact with each other and learn about and from each other in a social environment which eventually will influence their learning. Furthermore, the social constructivism theory emphasizes the zone of proximal development (17). This postulates that an individual will have skills/abilities they develop on their own but cannot perform them independently because they will need structured guidance from someone who has mastered the skill already to enable them to learn and be able to practice independently (17). Therefore, meaningful learning occurs when individuals are guided by experts utilizing a framework in a given field, engaged in social and collaborative activities within a community of practice such as that formed within an international elective placement, and optimally if done interprofessionally (18).

### Study aim

To address this gap, the purpose of this study was to develop an IPE framework guide for international electives to be used by health professions training institutions. Though the study was conducted in Sub-Saharan Africa, the framework developed can be adapted to various settings where international electives do occur.

## Methodology

### Setting

This study was conducted in Sub-Saharan Africa utilizing faculty and experts in representative health professions training institutions. The training institutions offer electives as part of their curriculum and conduct training for various health professional disciplines at the undergraduate level. Table 1 shows the details of the institutions and the respective expertise of the experts included in this study.

**Table 1:** Frequency distribution of the characteristics of the Delphi panel experts *N* = 8.

Variable	Frequency (*N*)
**Gender**	
Female	6
Male	2
**Discipline**	
Medicine	3
Nursing	2
Physiotherapy	1
Occupational therapy	1
Pharmacy	1
**Institutional affiliation**	
Busitema University, Uganda	1
Stellenbosch University, South Africa	2
University of Free State, South Africa/ African Interprofessional Network (AFRIPEN)	1
University of Global Health Equity, Rwanda/ African Forum for Research and Health (AFREhealth)	1
Makerere University Uganda/Yale University USA	2
University of Western Cape, South Africa	1
**Country location**	
Rwanda	1
South Africa	4
Uganda	1
United States of America (USA)	2
**Expertise**	
IPECP	3
International Elective placements	3
Health Professions Education	2

### Study design

In this study, we employed the modified Delphi technique to develop the IPE implementation framework for international electives in health professions training. In the Delphi approach, the first round could involve unstructured open-ended questions/ statements to guide the discussion with a panel of experts (19). When a set of structured selected questions/statements obtained from literature on the topic are used, this is called a modified Delphi technique and thus the approach used in this study (20). The Delphi technique is a structured process that uses a set of questions/ statements in various rounds with a panel of experts to gain consensus on a particular topic at hand (21). This technique was chosen because it is an appropriate method for topics with scanty evidence (22) such as IPE and its implementation in international electives in various training institutions where there is a lack of existing guidelines. It is also a method of choice just like in this study when there is a need to avoid individual opinions of the researcher dominating the process of seeking consensus across a wide expert panel. Furthermore, just like in this case, the Delphi method is an appropriate method of use when the panel of experts, who are the main participants are from various geographical locations which were key in this study (19). Many other researchers and educators have utilized the Delphi approach in developing frameworks and a consensus on various health agendas with an interprofessional approach (23–25). Given its variable success in other studies largely due to low response rate from experts, this technique was chosen in this study as the most appropriate technique to develop a framework to guide the implementation of IPE in international electives given the fact that it was to be done online and allowed each expert to select the dates and times that best work for them to participate.

### Informing the Delphi process

The Delphi process involved a series of stages before coming up with a final framework. These included conducting a rapid literature review, exploring opinions from students and faculty, and finally engaging the panel of experts for consensus building.

#### The rapid literature review (stage 1)

Firstly, a rapid literature review was conducted to inform the Delphi process for our study. This was done to enable a detailed understanding of IPE and international electives in line with the study objectives and to identify learning theories to be used. The rapid review was conducted using PubMed accessed through HINARI *via* Makerere University to identify literature on key concepts related to the topic under study. These included: internationalization in health professions education, international electives, the importance of IPECP in health care, current global disease burden, IPE during international electives, perceptions of faculty and students on IPE during international electives, IPE frameworks and their development, Learning theories for IPE, IPE competencies, the importance of IPE, the value of international electives, operational needs for international elective programs, teaching and assessment methods for IPE in international electives, and delivery approaches to teaching IPECP during international electives. The specific search terms used were: interprofessional education, international electives, interprofessional collaboration, Africa, IPE frameworks, Delphi technique, internationalization in health professions education, multidisciplinary, collaborative, interprofessional, interdisciplinary, teamwork, IPE teaching, assessment, theories, health professions education, and teaching.

We searched PubMed for manuscripts written in English, and from the years 2000 to 2022 as our inclusion criteria to enable us to have more articles included since there is scanty literature on this topic. The earliest year of the manuscript included was 2000 while the highest was 2021 with the majority being published in the last 10 years as shown in Supplementary Appendix 1. Papers that were just abstracts of a proposed study and did not report study findings were excluded. The rapid review approach provides more timely information for decision-making and is an appropriate method to inform the framework development using a Delphi approach (26). The rapid review was conducted by one researcher (FN). FN went ahead to develop a literature review write-up and shared it with IGM, AV, and AGM for review and appraisal. This approach and all the approaches used (one search engine, published papers, limiting inclusion criteria by language and date, one researcher (FN) conducting the review, and a senior team doing a secondary review and appraisal of the literature review findings) are acceptable approaches when conducting a rapid review for a consensus approach like the Delphi technique (26).

In this process, 84 manuscripts were identified and included (see Supplementary Appendix 1). The references in these papers were inspected for any additional evidence data and findings in line with this study’s aim. We conducted a framework analysis (27) to enable us to develop the literature review write-up from the rapid review. This enabled us to group articles into categories, themes, and narrative paragraphs in line with the study topic.

#### Seeking opinions from students and faculty (stage 2)

In addition to the rapid literature to inform the Delphi process, we sought opinions from students and faculty from four African health training institutions that participated in the study to understand their perceptions of IPE during international electives and their suggestions and views on an IPE framework for international electives. The students and faculty were given a brief orientation on what IPE is so that they could give meaningful responses related to IPE. The opinions and perceptions of the students and faculty were further utilized to enable the identification of some key constructs to be added to the draft framework that was used to build consensus. This study was conducted qualitatively among faculty using key informant interviews and quantitatively among students using an online survey. AtlasTI version 8 software was used for qualitative data analysis while SPSS IBM statistics 21 was used for quantitative data analysis. The various training institutions included: Makerere University Uganda, Kenyatta University Kenya, University of Ibadan Nigeria, and the University of Zimbabwe.

The specific details of the findings from the students have been published in the Journal of Interprofessional Care (28). The findings from the faculty have been accepted for publication in the Journal of Global Health Case Reports (29).

#### Engaging the panel of experts (stage 3)

The last stage was engaging the panel of experts to develop a draft framework. The draft framework was used to build consensus from the panel of experts and gain validation of the final framework.

### Draft framework used for consensus building

Through the rapid review, a Delphi guide developed by Bentley et al. (30) to develop a framework to implement IPE in primary health care was identified. Although this was used for primary health care, many international electives happen in the primary health care setting of the host institution country through clinical and community placements among others (31). Bentley et al’s Delphi guide further informed the refinement of the developed draft framework from stages 1 (rapid literature review) and 2 (student and faculty perceptions) before engaging the panel of experts for consensus building. It is this latter framework that was then used to seek and build consensus on various constructs of the IPE framework for international electives among the identified experts. The draft framework consisted of 8 sections as shown in Supplementary Appendix 2. These included; the relevance of IPE training in international electives in Africa, operational/organizational needs for IPE during international electives, acculturation needs, competencies to be gained by students participating in IPE international electives in Africa, IPE teaching approaches that can be utilized during international electives at host institutions, IPE assessment approaches during international electives at host institutions, mode of delivery and public health considerations. In the beginning, the public health consideration was labeled as COVID-19 precautions, but this later changed in the preceding rounds. In total, there were 52 statements over the eight sections. Each of these sections had various statements with two response options, i.e., agree and disagree to enable the experts to submit their views and guide consensus building.

#### Recruitment of the experts

Experts in IPECP, health professions education, and international electives were purposively sampled. Recruitment was from Makerere University and Busitema University in Uganda, Yale University USA, Stellenbosch University South Africa, University of Free state South Africa, University of the Western Cape, South Africa, and the University of Global Health Equity Rwanda. Some of the experts were members of the African Forum for Research and Education in Health, Ghana (one) and the African Interprofessional Network (one) while some (five) did not belong to any of these professional bodies in Africa. Furthermore, multidisciplinary representation was considered, and the experts were from various disciplines, i.e., medicine (three), nursing (two), pharmacy (one), occupational therapy (one), and physiotherapy (one). This enabled us to gain a heterogenous panel, a key requirement for the Delphi approach. In total, eight experts were recruited which is an acceptable number for the Delphi method. All eight members of the panel were academic experts in IPECP, health professions education, and international electives, with more than 5 years of experience as an academic faculty, and have had exposure to guiding and conducting international electives in Africa for more than 5 years.

### Sample size estimation for the panel experts

The Delphi method lends itself to the concept of the researcher assessing the scope of the problem and the available resources to determine the panel size that would be appropriate (19). Furthermore, it emphasizes that the researcher should consider the panel size depending on the experts’ skills and knowledge in the field, representation variability, experience, and work in the construct being studied (19). Thus, with the above guidelines on the selection of panel size, the number of experts included was 8 for this study. Furthermore, this number is within the acceptable panel size (8–1,685) for a Delphi method (32).

### The Delphi process

Engagement with the experts was done virtually due to their various geographical locations. The Delphi process involved three rounds. We began engagement with the panel of experts jointly as one group through email to generate interest and commitment. An email introduction to the 8 experts was done detailing the study aims, the process of participation, the time frame, etc. To ensure we capture full commitment to participate, a doodle poll was sent for all the experts to indicate their time of availability for Rounds 1 and 2. They were given 2 weeks to have this completed. All data collection and rounds were done online using Zoom meeting software for Rounds 1 and 2 (that were recorded), and email sharing for Round 3. Rounds 1 and 2 lasted one and a half hours while Round 3 lasted 3 weeks.

#### Round 1

For capturing the ratings of each expert on all 52 statements, the study tool was built in Microsoft Forms on an online survey platform. During the Zoom session, each of the participants was sent the online link. The researcher (FN) led the sessions and shared her screen to enable the experts to see the statements but also jointly go through them one by one as each of them submitted their responses using the online survey link as per the draft framework that was developed (Supplementary Appendix 2). This was done anonymously. Upon completion of Round 1, all the ratings and scores were in as shown in Table 2, and the researcher was able to access them in real-time. These were shared and projected to the experts online de-identified.

**Table 2:** Round 1 consensus on the IPE framework for international electives statements *N* = 8.

**Panel questions**	**Agree (%)**	**Disagree (%)**
**Relevance/Suggestions of IPECP training in international electives**		
1. Interprofessional training needs to be integrated across international elective placements in various health disciplines	75	25
2. More evidence is needed on the organizational and systemic facilitators, determinants, and barriers of interprofessional education for collaborative practice in international electives.	87.5	12.5
3. IPE in International Electives will foster efficient multinational teamwork and IPC among different countries, especially in epidemics and pandemics	50	50
4. It will lead to overall improved quality of health care at the patient, community, institution, and personal levels	50	50
5. This framework will guide students, faculty, institutional leaders, and Administration on how to effectively structure and implement IPE electives in Health Training Institutions	50	50
**Organizational/Operational needs**		
6. Committed home and host institutional leadership to supporting the program	100	0
7. Effective and well-oriented administrative support to lead students’ logistical needs and preparation	87.5	12.5
8. Committed IPE faculty at the host institution to support student learning and training	75	25
9. Effective agreements that allow reciprocity with home and host institutions	100	0
10. Learning facilities, infrastructure, and premises to aid student learning	62.5	37.5
11. Effective and clear application system in place to guide students on application requirements	62.5	37.5
12. Effective communication between home and host institution during preparations	87.5	12.5
13. Adequate financial support to cater to logistical costs	100	0
14. Students from 2 or more different professional disciplines from home and host institutions (preferably those in the clinical training years)	62.5	37.5
15. The IPE student groups during the elective placement at host institutions should include a minimum of 2 or more disciplines	75	25
16. Each IPE student group during the elective should have 2–8 students to enable adequate learning	62.5	37.5
**Acculturation needs**		
17. Effective Pre elective orientation courses/ workshops offered to students, faculty, clinical and community instructors, and administrators to enable effective understanding of roles, expectations, and the domains of IPEC training, culture, setting, and flow of activities	62.5	37.5
18. Onsight Orientation by the admin and supervising faculty	62.5	37.5
**Defining and understanding Competencies to be gained** **This section covers knowledge, competencies, capabilities for interprofessional education and training during international electives in any discipline of choice.**		
19. Interprofessional learning outcomes relating to teamwork, i.e., Knowledge of, and skills for, teamwork	87.5	12.5
20. Interprofessional learning outcomes related to roles and responsibilities, i.e., Knowledge and understanding of the different roles, boundaries, responsibilities, and expertise of health professionals.	75	25
21. Being able to challenge misconceptions in relation to roles	25	75
22. Interprofessional learning outcomes related to communication, i.e., Ability to communicate effectively with other health professional students	62.5	37.5
23. Awareness of cultural differences in health profession command and conduct in another country	87.5	25
24. Ability to express one’s opinions with others involved in patient care	87.5	12.5
25. Interprofessional learning outcomes relating to learning/reflection, i.e., Ability to reflect critically and evaluate their performance and that of the team	87.5	12.5
26. Ability to transfer interprofessional learning gained during the international elective back home in the clinical, community, or public health setting	87.5	12.5
27. Interprofessional learning outcomes relating to the patient/client, i.e., Ability to recognize the central role of the patient in collaborative care	87.5	12.5
28. Interprofessional learning outcomes relating to ethics/attitudes, i.e., Ability to acknowledge the views and ideas of other professionals during an international elective placement	100	0
29. Understanding the ethical issues relating to teamwork	87.5	12.5
**IPECP Teaching Approaches that can be utilized during international electives**		
30. Simulation-based IPE training	87.5	12.5
31 Observership-based interprofessional learning.	50	50
32. Team-based approaches during clinical ward rounds and bedside teaching	100	0
33. Community placements with local students from various health disciplines	100	0
34. Case study-based interprofessional learning with local students	87.5	12.5
35. Lecture/seminar-based education and training sessions	37.5	62.5
**IPEC learners assessment approaches during international electives**		
36. Pre-post course knowledge/skills/attitude surveys	100	
37. Peer-to-peer Assessment	100	
38 Self-assessment/reflection (metacognitive skills) (Elective Report)	75	25
39. Portfolio-based assessments (collection and review of individual and group work projects or assignments done)	100	
40. Team Observed Structured Clinical Examination (TOSCE)	75	25
41. Simulated cases involving inter-professional practice	87.5	12.5
42. Group feedback sessions	75	25
**Mode of elective delivery**		
43. Online utilizing the teaching and assessment approaches that can be applied in a virtual platform, e.g., country-specific case studies	87.5	12.5
44. Actual outbound mobility physical mobility to a specific host institution	75	25
45. Blended approach with both online and actual mobility	100	0
**COVID 19/considerations**		
46. Adherence to the public health guidelines for home and host institutions	100	0
47. The blended model can be used	100	0
48. Negative COVID-19 tests before and after elective placement	75	25
49. Vaccination is mandatory before rotation	75	25
50. Wearing of PPE and hand sanitization always	87.5	12.5
51. Fewer cohorts of students hosted at a time	37.5	62.5
52. Social distancing in all activities	37.5	62.5

Consensus was at ≥ 70% per statement if the panel selected agree.

Globally, there is a lack of uniform guidelines on what constitutes consensus in a Delphi study (21). Different studies use different approaches that suit them best (21). Three consensus measurements have been used by various studies (21). These include percentage level of agreement, median scores, and interquartile ranges (21). Because the statements in the tool had two options to establish agreement, i.e., agree and disagree, and no scores *per se* or Likert scale, we used a percentage level of agreement to establish consensus. A score of ≥70% on a particular statement was deemed as relevant and important to be included in the framework while a ≤ 69% score on any statement meant that there was a lack of consensus and thus needed to be discussed and taken up for Round 2. This percentage level of agreement and disagreement was adopted from existing literature from studies that have used the Delphi process for health issues consensus building.

#### Round 2

During Round 2, participants were given the scores on all statements, i.e., those that achieved consensus and those that did not. Participants were invited to comment, discuss, and rate the 15 statements that did not reach consensus, i.e., statements with scores of ≤69%. Each of the participants was given a chance to discuss and give their views on these statements and all the suggestions were captured by the researcher. An agreement was reached to leave out the section on the importance of international electives in Africa. This is because the framework is meant to guide the implementation of IPE during international electives and the importance of IPE is already well elaborated from the various existing frameworks and literature (1). The COVID-19 consideration section was condensed into one statement and the name changed to Public Health Considerations. This is because health considerations go beyond COVID-19 and thus it makes it more relevant to adhere to the host country’s public health considerations at the time one participates in the international elective. The statement on misconceptions clarification in the section for IPE competencies and lectures in the section on IPE teaching during international electives was dropped. The section on IPE assessment was subdivided into two sections to reflect formative and summative assessment methods. The section on acculturation was split into 3 statements instead of one. Furthermore, all statements that met the agreement score were also discussed for better wording and presentation.

Upon completion of Round 2, all comments and suggestions from Round 2 coupled with overall suggestions on all statements including those that achieved consensus were taken into consideration. All the changes were made, and a revised version of the draft revised framework was shared with the experts for a rating in round 3. While Round 1 had 52 statements, upon revision and consideration of all suggestions from Round 2, the framework had 42 statements to be used for Round 3.

#### Round 3

Round 3 involved sharing the revised version of the statements with the experts via email. Supplementary Appendix 3 shows the revised framework used for Round 3 consensus building. Each of them was allowed to rate and give their view on each statement within 3 weeks. Table 3 shows the scoring for Round 3 from all the experts who responded. After this, all statements had reached a consensus and thus the revised framework was organized into a final framework format with grammatical edits made. The experts were also given a chance both in Rounds 1 and 2 to share their ideas on practical guidance for the implementation of each of the statements. These points were captured by the researcher and developed into section 3 as a practical guide on how to implement the developed framework. The final framework format was shared with the experts one more time for final review and validation. All responses or scores were analyzed within MS forms, automatically calculating the percentage scores.

**Table 3:** Round 3 consensus on the IPE framework for international electives Delphi guide statements *N* = 8.

**Home and host training institution’s operational needs for IPE during international electives**	**Agree (%)**	**Disagree (%)**
1. Home and host institutional leadership support for IPE in international electives programs	100	
2. Home and host institution administrative support to handle students’ logistical needs before, during, and after the IE placement	100	
3. Faculty trained in IPE at the host institution to support and supervise students	87.5	12.5
4. Partnership agreements that explore and allow reciprocity with home and host institutions	100	
5. Learning facilities to aid student learning	75	25
6. Clear application system in place to guide students on IPE elective application requirements	100	
7. Communication strategy between home and host institution during preparations, implementation, and post-participation	100	
8. Adequate financial support to cater to students’ logistical costs	100	
9. Students from 2 or more different professional disciplines from home and host institutions (preferably those in the clinical training years)	87.5	12.5
10. The IPE student groups during the elective placement at host institutions should include a minimum of 2 or more disciplines	87.5	12.5
11. Each IPE student group during the elective should have 2–8 students to enable adequate learning	87.5	12.5
**Acculturation considerations**		
12. Pre-elective IPE orientation didactic sessions or seminars offered by the host institution to students, to enable understanding of roles, expectations, the domains of IPE, and the flow of activities	100	
13. Pre-Elective IPE training (workshops or seminars) offered to faculty, clinical and community instructors, to enable understanding of roles, expectations, the domains of IPE, and the flow of activities	87.5	12.5
14. Onsite Orientation by the host institution on various social aspects and living to enable acclimatization of students in consideration of language, cultural humility, and equity.	87.5	12.5
**Competencies to be gained by students participating in IPE international electives.** **By the end of the international elective students should be able to;**		
15. Demonstrate Knowledge attitudes, and skills for, teamwork	100	
16. Demonstrate knowledge and understanding of the different roles, boundaries, responsibilities, and expertise of various health professionals in the team	100	
17. Communicate effectively and respectfully with other health professionals’ students, faculty, patients, community, etc.	100	
18. Demonstrate an awareness of cultural differences in health profession command and conduct in another country	100	
19. Express one’s opinions with others involved in patient care with respect and humility	100	
20. Reflect critically and evaluate their performance and that of the team	100	
21. Develop a plan on how to apply interprofessional education and skills gained during the international elective back home in the clinical, community, or public health setting	100	
22. Recognize the central role of the patient/ community in collaborative care	100	
23. Acknowledge the views and ideas of other professionals during an international elective	100	
**IPE teaching approaches that can be utilized during International Electives at Host institutions**		
24. Simulation-based IPE teaching	87.5	12.5
25. Interprofessional community placements	100	
26. Country-specific case study-based interprofessional teaching	100	
27. Joint tutorials using a flipped-classroom approach	100	
28. Joint clinical placements through joint ward rounds and bedside teaching	100	
**IPE learner’s assessment approaches during international electives at host institutions**		
**Formative (ongoing assessment)**		
29. Pre-elective course knowledge/skills/Attitudes Surveys	100	
30. Portfolio-based assessments (collection and review of individual and group work projects or assignments done)	87.5	12.5
31. Simulated cases involving interprofessional practice	100	
32. Peer to Peer assessment	75	25
33. Team Observed Structured Clinical Examination (TOSCE)	100	
**Summative assessment (end of program assessment)**		
34. Post Elective course knowledge/skills/attitude surveys	100	
35. Self-reflection through Elective Report at the end	87.5	
36. Team Observed Structured Clinical Examination (TOSCE)	100	
37. Simulated cases involving interprofessional practice	100	
38. Group feedback sessions	75	25
**Mode of elective delivery**		
39. Online: utilizing the teaching and assessment approaches that can be applied in a virtual platform, e.g., country-specific case studies	87.5	12.5
40. Actual outbound physical mobility to a specific host institution	100	
41. Blended approach with both online and actual mobility at the host institution	100	
**Public health considerations**		
42. Adherence to the public health national guidelines for home and host institutions and countries with respect to health and safety requirements for traveling trainees.	100	0

Consensus was at ≥ 70% per statement if the panel selected agree.

## Results

This section describes the key outcomes of the Delphi process undertaken to develop an IPE framework during international electives for various African training institutions. We had all eight experts fully participating in all the rounds, thus a 100% response rate. The details and characteristics of the Delphi panel of experts are displayed in Table 1.

The results from Round 1 and Round 3 are displayed in Tables 2, 3 respectively. The results and outcomes of Round 2 are presented as a narrative. The final framework developed is described and attached in Supplementary Appendix 4.

Round 2 involved discussions on statements in Round 1 that had not reached a consensus. Furthermore, there were suggestion on how to phrase the statements that had reached a consensus. The actual changes made have been described in the data collection process section for Round 2 above. All these revisions were made, and Supplementary Appendix 3 shows the revised framework developed for use in Round 3 for consensus building.

### The developed IPE framework

The key outcome of this study is the IPE framework (Supplementary Appendix 4). IPE Framework that has been developed. This framework is illustrated in Supplementary Appendix 4. The framework aims to guide health education institutions on how to incorporate and implement IPE in international electives for health and allied health programs. The framework begins by mentioning its intended aim and a clear definition of the terms being used. The framework consists of three sections. Section 1 highlights the various IPECP competencies to be gained by learners in the areas of teamwork, interprofessional communication, roles, and responsibilities of interprofessional collaborative practice, values, and ethics of interprofessional collaboration, and reflection and evaluation of oneself and the team. Section 2 gives guidance on the structuring of the IPE international electives in health professional training institutions. This includes subsections on operational/institutional needs, acculturation considerations, teaching strategies, assessment strategies, mode of delivery, and public health considerations. Section 3 consists of the various practical guides and approaches that health professional training institutions could use according to what works best in their setting. Both home and host institutions should be able to utilize the framework to enable a well-structured international elective.

## Discussion

We set out to develop a framework to guide IPE training during international electives in various health professions training institutions in Sub-Saharan Africa. With the use of a modified Delphi approach, we were able to develop an IPE framework for international electives adaptable to various settings including Africa. The modified Delphi approach allowed the appreciation of the social constructivism theory in relation to various environments shaping one’s knowledge and learning. This method allowed us to have experts from various locations jointly interact and share their perspectives from their context and harmonize what would eventually lead to structured IPE international electives through a framework to guide learning for the students and the experts that deliver the learning. This also aligned with the aspects of the zone of proximal development as per the social constructivist theory. International electives are learning environments that are key to the development of global perspectives on various disease burdens and approaches to addressing them (33). International electives are also a key ground to cultivate various approaches to healthcare delivery for enhanced patient outcomes while gaining more global exposure to articulate clinical knowledge and skills (34). Given their relevance, a drive to have International electives structured with innovative approaches has been ongoing to enable meaningful engagement for students with transformative learning experiences (3).

IPE is an innovative approach to international electives and another key ground that can be used to foster IPECP (35). However, globally and in Africa, there is hardly any framework to guide training institutions to implement IPE during international electives. The majority of the international electives occur in silos with most of the students mainly exposed to the faculty of their disciplines and only rotating with students of similar disciplines (33). Various training institutions have international offices that could be used as a ground for innovation in health professions education (36). However, these are mainly administrative and handle the needs of incoming and outgoing elective students (36). To address this gap and steer the momentum for IPE during international electives among various training institutions, we developed a framework to guide the implementation of IPE during international electives using a modified Delphi approach. This approach was adequate to enable us to build consensus on the various parameters and domains. The framework can enable institutions and faculty to structure IPE international electives feasibly. The Delphi approach usually has been reported to have various limitations that include a drop-off in participation by the experts, self-selection of participants, researcher bias, and non-responder bias (37). However, in this study, we had a high response rate with all the experts actively participating in all rounds. This can be attributed to the fact that we had a manageable low number of experts (38), who are very well experienced (39) in all domains of the topic, coupled with an online approach that enabled each of them to indicate their availability and thus allowing us to fit in the experts’ schedules.

A high consensus mark of ≥70% was used for addressing the aspects of validity. This mark (≥70%) has been used in other studies to build consensus in various health professions education research including IPE (30). Many times, various studies in health professions education and IPE that have used the Delphi approach have used Likert scales for consensus building among the experts (21). However, at the time of computing consensus, to enable a unified understanding of agreement and disagreement, the various Likert categories are often grouped into two, i.e., agree and disagree (30). It is on this premise that we used two options for consensus building, i.e., agree and disagree for all the statements and constructs to avoid any confusion. Furthermore, Round 2 enabled each panel expert to explain and seek clarification on any statement thus being able to make an informed decision of agreement or disagreement. Furthermore, the online approach (despite differences in time zones and geographical location) is a key strength in enabling experts to meet and actively participate thus addressing the usual limitations of Delphi through asynchronous correspondence. A study done by Donohoe et al. (40) has shown the strength of an online approach as key in addressing the nonresponse limitation. Furthermore, the online approach enables the effective building of consensus through timely consensus score submission. It has been used in this study and similarly in the development of the IPE framework to guide IPE in primary health care (30).

In this study, we had representation from various geographical locations, disciplines, and expertise which enabled triangulation and adaptability in various training institutions including Africa (21). Although the framework was developed in Sub-Saharan Africa using the modified Delphi approach, it can be used in other international electives settings even beyond Africa where interprofessional education is being sought.

The IPE core competencies of this framework were tailored toward gaining IPECP skills that can be applied in all areas of healthcare practice for the students in their future practice. These competencies in our framework were adapted as a combination of various IPECP competencies developed by the European Interprofessional Practice and Education Network (6), the Interprofessional Education Collaborative (4), and the Center for the Advancement of Interprofessional Education (41). This allowed us to compare each of them, identify the strengths of each, and utilize them to develop competencies that are appropriate for an international elective learning environment. This approach has not only been used in this study but in other Delphi approaches that have attempted to develop structure around IPE in health care and training (30).

Structuring any new approach in health professions education involves various levels of preparation. This includes the leadership, administration, teaching faculty, training facilities, teaching and assessment methods, public health safety considerations, and modes of delivery among others. Section 2 of the framework covers this in detail. It gives guidance and approaches on how to enable leadership support for IPE during international electives. For international electives, this should be done through institutional partnerships with home and host institutions. Our framework emphasizes reciprocity to enable equity which is key in building equitable partnerships for training, especially in international electives where there is often an imbalance in opportunities for students from various African training institutions (8). In this framework, we emphasize ensuring that all academic faculty that would be involved in IPE in international electives are trained on IPECP. This is because various studies in Africa have shown a significant gap among faculty on IPECP (42). This can be done through online or in-person workshops that are focusing on the various core competencies and principles of IPE, the importance of IPE in health care, approaches to teaching, and assessment coupled with the mode of delivery. This in the long run enables faculty to gain the skills on how to handle training for students from various healthcare disciplines and capitalize on interprofessional student teams achieving the various IPE learning outcomes during the international electives.

The teaching and learning approaches put across as options in this framework have been described in the literature that was used to inform this process. In particular small group learning (i.e., 2–8 students) has been efficient in various approaches to group training (43). This, therefore, means that for efficient IPE training during international electives, this should be done in small groups to enable faculty to pay attention to each of the students, coupled with enabling maximum interaction of the students with each of the group members. Furthermore, this number still falls in the prescriptions of interprofessional team composition (i.e., 2 or more healthcare professionals from various care disciplines) (44).

Utilization of bedside teaching (45), clinical placements (46), community placements (47), simulation (48), case studies (49), and tutorials (50) are well-established training approaches in various healthcare training institutions in Africa and thus forming a premise of methods that are applicable and available to be used for IPE during international electives depending on each institution’s resources available. This, therefore, means that faculty have a wide range of choices based on the various methods they use for their regular teaching to utilize for IPE international electives. Lectures are deemed a very important method or approach of training in health professions education globally (51). However, in this framework, this is not listed as a form of training to be used to teach IPE during international electives. This is because the expert’s view was that IPE requires practical involvement to essentially gain the IPECP skills. Lectures, therefore, in this framework were deemed appropriate to be under acculturation to enable the faculty to articulate the core principles and competencies of IPE to the students and thus in the long run give them an orientation on the IPECP concepts. This can be done through (online or physical) lectures which are efficient in providing an overview and knowledge of the IPE core competencies and not necessarily enabling students to gain IPECP skills (52).

Clinical observerships as a form of training commonly used in international electives in developed countries (53) did not reach a consensus of agreement in Round 1. In Round 2 this was dropped and not included in Round 3. This is because the panel of experts ascribed gaining of IPECP skills to practical engagement which observerships lack. This, therefore, means that any IPE teaching methods like those described in the framework we have developed should have avenues for practical student engagement activities.

Assessment of acquisition of IPECP competencies during international electives should be formative and summative as exhibited in this framework to capture the various IPECP skills gained by the students as they participate in an IPE international elective. Pre and Post-elective surveys have been popularly used to establish the IPECP gained (54). In the practical guide of the framework regarding assessment tools, i.e., pre and post-participation surveys, the Interprofessional Collaborative Competencies Attainment Scale 2018 (ICCAS 2018) (55) is recommended for assessment of the attainment of the IPECP before and after the elective. This is because this scale allows scoring and has a guide on the interpretation of the total scores and categories and what they mean for the learner and the faculty performing the assessment. Furthermore, the behavioral indicators of interprofessional practice assessment tool by EIPEN is one of the most recent tools developed to ascertain interprofessional practice. In this study, this tool is being recommended for long-term assessment of IPECP given that behavioral change toward interprofessional practice needs ample time to measure. The other approaches of assessment (i.e., peer to peer assessment, report writing, and team-based objective structured exams) are those that have been used widely in IPE and encourage student team participation (56). To a larger extent, they encourage student-led assessment which often is key in enabling a learner to have a reflection on themselves and that of the team during IPE international electives an important competency for interprofessional collaboration (6).

Globally various modes of delivery have been used to deliver IPE. These include the physical or in-person approaches that involve face-to-face interaction of interprofessional teams of students with faculty (57), and the blended approach that has both online and in-person interaction (58). Although the latter are key approaches that can also be utilized in international electives, what has recently picked momentum is the virtual mode of delivery of international electives (59), mainly accelerated by the COVID-19 pandemic that has enhanced the use of the internet and digital platforms in health professions education even in Africa (60). This, therefore, means that depending on the resources and the information technology systems available at various institutions, the virtual mode of delivery for IPE during international electives can be done utilizing platforms like Zoom or MS Teams, which are widely used in various training institutions. This can be through country-specific case studies, joint tutorials, and discussions all guided by IPE-trained faculty (61). This in the long run could be an approach that makes international electives even much more cost-effective given the elimination of various travel and accommodation costs required for physical and blended approaches of international electives. It is key to note that this requires reliable internet which despite its hardships in Africa has been steadily enhanced in various African countries (62) since the COVID-19 pandemic (60).

## Conclusion

The modified Delphi technique was an adequate approach to aid the development of an IPE framework to guide implementation during international electives in various health professional training institutions. The IPE framework developed is adaptable and can be implemented in various health professional training institutions. The framework developed enables effective structuring of IPE international electives given that it has all domains required in implementing international electives, i.e., the core competencies to be gained, the operational institutional needs, teaching and assessment methods, modes of delivery, and public health considerations, especially for the fact that international electives require mobility to another country. Unlike other frameworks, this framework also provides some practical guidance on various approaches to implementing IPE during international electives thus creating a benchmark for new approaches to learning during international electives, especially in low-income settings.

IPE is one of the key health professions education research priorities for sub-Saharan Africa (51). Our work to a large extent contributes to this agenda but also creates a new platform for more research by the users of our framework by various institutions, in various health specialties. Structuring IPE in health professional training institutions is possible and our framework creates a starting point for this specifically, in international electives.

### Recommendations

The modified Delphi approach is an adequate approach to be used to develop IPE frameworks to support IPE training in various learning environments including international electives. With the framework now in place to guide the implementation of IPE in international electives, a pilot has been done in the four institutions that participated in this study’s Delphi information process. The results have been published by BMC Medical Education (63). However, there is a need for more institutions to pilot its use and document their findings to enhance its validity.

### Quality control

Given the qualitative nature of the Delphi approach, trustworthiness and rigor were observed. For credibility, prolonged engagement with the experts, various rounds to enable consensus, having a cut-off point for consensus, review of the final framework by the experts were done. To ensure triangulation the use of both a research study and rapid literature review to inform the process, experts from various training institutions, and disciplines was done. A detailed description of the methods used was done to enable transferability in similar contexts. To ensure the dependability of the findings various rounds with scores to establish consensus were done. Lastly, confirmability was observed by having the final framework reviewed by the study team and the panel of experts for accuracy and alignment with the study objectives.

### Limitations and strengths

The panel of experts was in different countries and time zones thus leading to the use of online options to conduct all the other rounds. However, despite the online virtual approach, we were able to record the sessions thus enabling the replay of the sessions for point articulation. Furthermore, the online approach enabled timely rating of the statements which allowed a quick turnaround time. The number of experts involved may be seen as a limitation to some however, this was still within the acceptable number for a Delphi panel recommended (8–1,685) (38). Four institutions were involved in seeking opinions from the students and faculty. These were few compared to the number of health professional training institutions in Sub-Saharan Africa. However, it is key to note that the institutions selected were those that provided credit for the participation of international electives and were in the various cardinal regions of Africa. The number of faculty and students may be seen as few however the number of those involved was arrived at using scientific sample size calculation for the students and point of saturation for the faculty given that it was a qualitative study. One of the strengths of this study is the consideration of experts from various disciplines, geographical locations, and with experience in IPE, health professions education, and international electives. This thus enabled the development of a framework that can be applied in various settings in Africa and promote IPE among various health professional disciplines during international electives. Furthermore, the consensus-building approach enabled the elimination of any researcher bias as consensus was dependent on the full panel and not the research alone.

## Data availability statement

The original contributions presented in the study are included in the article/Supplementary material, further inquiries can be directed to the corresponding author.

## Ethics statement

This study followed all the required regulations and guidelines by the Helsinki Declaration. Ethical approval to conduct the study was granted by the Makerere University School of Medicine Research Ethics Committee (SOMREC) Mak-SOMREC-2021-96 and the Uganda National Council for Science and Technology (UNCST) HS2078ES. Administrative clearance was obtained from the University of Ibadan, the University of Zimbabwe, and Kenyatta University. Written Informed consent was sought from all participants of the study.

## Author contributions

FN, IM, AV, SK, and AM conceptualized and developed the study and jointly drafted the manuscript. FN implemented the study. FN, IM, and AM cleaned and analyzed the data. TR and FW contributed to the final review and development of the framework developed in this study.
