# Supplementary material for: Using the modified Delphi technique to develop a framework for interprofessional education during international electives in health professions training institutions in Sub-Saharan Africa
Source: Front Med (Lausanne). 2023 Oct 18;10:1225475. doi: 10.3389/fmed.2023.1225475 (PMC10618419; doi:10.3389/fmed.2023.1225475)
Supplement: Supplementary file 1 [file Data_Sheet_1.PDF]

Appendix 1: List of Manuscripts Included in the Rapid Review to Inform the Delphi Process for the Development of the IPE framework for International Electives in African Health Professional Training Institutions

1. Knight J. Internationalization Remodeled: Definition, Approaches, and Rationales. 2004. https://journals.sagepub.com/doi/10.1177/1028315303260832.

2. Hudzik J. Comprehensive Internationalization Institutional pathways to success. Routledge; 2014.

3. Leask B. Internationalizing the Curriculum. London: Routledge; 2015.

4. LeBeau LG. A Process Approach to Internationalization--Utilizing De Wit's Internationalization Circle (Modified Version) for Internationalization Planning. International Research and Review. 2018;7:1–17.

5. Altbach P. Global Perspectives on Higher Education. Baltimore. 1st edition. Baltimore: The Johns Hopkins University Press; 2016.

6. Jowi JO, Knight J, Sehoole C. Internationalisation of African Higher Education. In: Sehoole C, Knight J, editors. Internationalisation of African Higher Education: Towards Achieving the MDGs. Rotterdam: SensePublishers; 2013. p. 11–31.

7. Wan X, Geo-JaJa MA. Internationalisation of Higher Education in Africa: Characteristics and Determinants. World Studies in Education. 2013;14:79–101.

8. Wu A, Leask B, Choi E, Unangst L, de Wit H. Internationalization of Medical Education—a Scoping Review of the Current Status in the United States. Med Sci Educ. 2020;:1–13.

9. Wu A, Noel G. How to Internationalize Medical Education using Concepts in Internationalization of Higher Education. MedEdPublish. 2020;9.

10. Koplan JP, Bond TC, Merson MH, Reddy KS, Rodriguez MH, Sewankambo NK, et al. Towards a common definition of global health. The Lancet. 2009;373:1993–5.

11. Battat R, Seidman G, Chadi N, Chanda MY, Nehme J, Hulme J, et al. Global health competencies and approaches in medical education: a literature review. BMC Medical Education. 2010;10:94.

12. Grudzen CR, Legome E. Loss of international medical experiences: knowledge, attitudes and skills at risk. BMC Med Educ. 2007;7:47.

13. Muir J, Farley J, Osterman A, Hawes S, Martin K, Stephen Morrison J, et al. Global Health Programs and Partnerships: Evidence of Mutual Benefit and Equity. 2016.

14. Centre for International Mobility CIMO. North–South–South 10 Years: A Decade of Supporting Development Through Academice Mobility. 2015.

15. Law IR, Worley PS, Langham FJ. International medical electives undertaken by Australian medical students: current trends and future directions. Med J Aust. 2013;198:324–6.

16. Cantu MP. Three Effective Strategies of Internationalization in American Universities. Journal of International Education and Leadership. 2013;:12.

17. Olu O, Petu A, Ovberedjo M, Muhongerwa D. South-South cooperation as a mechanism to strengthen public health services in Africa: experiences, challenges and a call for concerted action. Pan Afr Med J. 2017;28.

18. Dowell J, Merrylees N. Electives: isn't it time for a change? Med Educ. 2009;43:121–6.

19. Crump JA, Sugarman J, Working Group on Ethics Guidelines for Global Health Training (WEIGHT). Ethics and best practice guidelines for training experiences in global health. Am J Trop Med Hyg. 2010;83:1178–82.

20. TIMS. How much will a clinical elective in the US cost? The Indian Medical Student. 2019. https://theindianmedicalstudent.com/how-much-will-a-clinical-elective-in-the-us-cost/.

21. Nyongesa H, Mokua W, Adegu J. Medical trainees' experiences and perceptions towards elective period; a cross sectional study. Pan Afr Med J. 2014;17.

22. Abedini NC, Danso-Bamfo S, Moyer CA, Danso KA, Mäkiharju H, Donkor P, et al. Perceptions of Ghanaian medical students completing a clinical elective at the University of Michigan Medical School. Acad Med. 2014;89:1014–7.

23. Bozinoff N, Dorman KP, Kerr D, Roebbelen E, Rogers E, Hunter A, et al. Toward reciprocity: host supervisor perspectives on international medical electives. Medical Education. 2014;48:397–404.

24. Johnston N, Sandys N, Geoghegan R, O'Donovan D, Flaherty G. Protecting the health of medical students on international electives in low-resource settings. J Travel Med. 2018;25.

25. Fotheringham EM, Craig P, Tor E. International medical electives in selected African countries: a phenomenological study on host experience. Int J Med Educ. 2018;9:137–44.

26. Bozinoff N, Dorman KP, Kerr D, Roebbelen E, Rogers E, Hunter A, et al. Toward reciprocity: host supervisor perspectives on international medical electives. Med Educ. 2014;48:397–404.

27. Kumwenda B, Royan D, Ringsell P, Dowell J. Western medical students' experiences on clinical electives in sub-Saharan Africa. Medical Education. 2014;48.

28. Rajeswaran L. Clinical Experiences of Nursing Students at a Selected Institute of Health Sciences in Botswana. Health Science Journal. 2016;10.

29. Stetten NE, Black EW, Edwards M, Schaefer N, Blue AV. Interprofessional service learning experiences among health professional students: A systematic search and review of learning outcomes. Journal of Interprofessional Education & Practice. 2019;15:60–9.

30. Willott C, khair E, Worthington R, Daniels K, Clarfield AM. Structured medical electives: a concept whose time has come? BMC Globalization and Health. 2019. https://globalizationandhealth.biomedcentral.com/articles/10.1186/s12992-019-0526-2#Sec1.

31. CDC. Major Partners Involved in CDC's Response to the 2014-2016 Ebola Epidemic. 2019. https://www.cdc.gov/vhf/ebola/outbreaks/2014-west-africa/partners.html.

32. World Health Organization, World Health Organization. 2015 WHO strategic response plan: West Africa Ebola outbreak. Geneva: World Health Organization; 2015.

33. DuBois M, Wake C, Sturridge S, Bennett C. The Ebola response in West Africa. Exposing the politics and culture of international aid. Humanitarian Policy Group Overseas D203 Blackfriars Road London SE1 8NJ United Kingdom: Humanitarian Policy Group. Overseas Development Institute; 2015.

34. Mohamed K, Rodríguez-Román E, Rahmani F, Zhang H, Ivanovska M, Makka SA, et al. Borderless collaboration is needed for COVID-19—A disease that knows no borders. Infect Control Hosp Epidemiol. 2020;:1–2.

35. WHO. Framework for Action on Interprofessional Education & Collaborative Practice. 2010;:64.

36. Hugh B. Interprofessional Education Guidelines. CAIPE. 2017. https://www.caipe.org/resources/publications/caipe-publications/barr-h-gray-r-helme-m-low-h-reeves-s-2016-interprofessional-education-guidelines.

37. van Diggele C, Roberts C, Burgess A, Mellis C. Interprofessional education: tips for design and implementation. BMC Medical Education. 2020;20:455.

38. Green BN, Johnson CD. Interprofessional collaboration in research, education, and clinical practice: working together for a better future. The Journal of Chiropractic Education. 2015;29:1.

39. Baily K. IPE vs. Multidisciplinary Education: What's the Difference for Clinical Simulation (w/ Downloadable Tool) | HealthySimulation.com. 2020. https://www.healthysimulation.com/22475/ipe-healthcare-simulation/.

40. Epstein RM, Hundert EM. Defining and assessing professional competence. JAMA. 2002;287:226–35.

41. Birk TJ. Principles for Developing an Interprofessional Education Curriculum in a Healthcare Program. Journal of Healthcare Communications. 2017;2.

42. Merriman C, Chalmers L, Ewens A, Fulford B (KWF), Gray R, Handa A, et al. Values-based interprofessional education: how interprofessional education and values- based practice interrelate and are vehicles for the benefit of patients and health and social care professionals. Journal of Interprofessional Care. 2020;34:569–71.

43. Reeves S. Why we need interprofessional education to improve the delivery of safe and effective care. Interface (Botucatu). 2016;20:185–97.

44. IPEC. Core Competencies for Interprofessional Collaborative Practice: 2016 Update. 2016;:22.

45. EIPEN. The EIPEN Key Competences for Interprofessional Collaboration. 2020.

46. Bennett B, Carney T. Public Health Emergencies of International Concern: Global, Regional, and Local Responses to Risk. Med Law Rev. 2017;25:223–39.

47. Daniels K, Thomson E, Nawagi F, Flinkenflögel M. Value and feasibility of South-South Medical Elective Exchanges in Africa. BMC Med Educ. 2020;20:319.

48. Wiskin C, Dowell J, Hale C. Beyond ‘health and safety’ – the challenges facing students asked to work outside of their comfort, qualification level or expertise on medical elective placement. BMC Med Ethics. 2018;19:74.

49. Nawagi F, Iacone AM, Tinder D, Seeling J, Kamami E, Sit A. African Forum for Research in health (AFREhealth) student exchange pilot: an impact assessment. The Lancet Global Health. 2019;7:S28.

50. University of Cape Town. About Clinical Elective Placements | International and local electives. 2021. http://www.healthelectives.uct.ac.za/about/elective-placements.

51. Flinkenflögel M, Essuman A, Chege P, Ayankogbe O, De Maeseneer J. Family medicine training in sub-Saharan Africa: South–South cooperation in the Primafamed project as strategy for development. Fam Pract. 2014;31:427–36.

52. Muir A Jonathan, Farley Jessica, Osterman Allison, Hawes Stephen, Keith Martin, Marrison J Stephen, et al. Global Health Programs and Partnerships. Evidence of Mutual Benefits and Equity. CSIS Global Health Policy Center and University of Wshington Global Health STAT Center; 2016.

53. Law IR, Walters L. The influence of international medical electives on career preference for primary care and rural practice. BMC Med Educ. 2015;15:202.

54. Mahler C, Schwarzbeck V, Mink J, Goetz K. Students´ perception of interprofessional education in the bachelor programme “Interprofessional Health Care” in Heidelberg, Germany: an exploratory case study. BMC Medical Education. 2018;18:19.

55. Alzamil H, Meo SA. Medical students’ readiness and perceptions about Interprofessional Education: A cross sectional study. Pak J Med Sci. 2020;36:693–8.

56. Quartey J, Dankwah J, Kwakye S, Acheampong K. Readiness of allied health students towards interprofessional education at a university in Ghana. African Journal of Health Professions Education. 2020;12:86–9.

57. Byakika P, Kutesa A, Baingana R, Muhumuza C, Kitutu FE, Mwesigwa C, et al. A situation analysis of inter-professional education and practice for ethics and professionalism training at Makerere University College of Health Sciences. BMC Res Notes. 2015;8:598.

58. Botma Y, Labuschagne M. Students' perceptions of interprofessional education and collaborative practice: analysis of freehand drawings. Journal of Interprofessional Care. 2019;33:321–7.

59. Hinderer KA, Klima D, Truong H-A, Rangel AG, Brown V, Talley W, et al. Faculty Perceptions, Knowledge, and Attitudes Toward Interprofessional Education and Practice. J Allied Health. 2016;45:e1-4.

60. Bennett, Gum L, Lindeman I, Lawn S, McAllister S, Richards J, et al. Faculty perceptions of interprofessional education. Nurse Education Today. 2011;31:571–6.

61. Chitsulo C, Chirwa E, Wilson L. Faculty knowledge and skills needs in interprofessional Education among faculty at the College of Medicine and Kamuzu College of Nursing,. Malawi Medical Journal. 2021.

62. Ross F. World Health Organization Announcement. Journal of Interprofessional Care. 2007;21:587–9.

63. de Villiers MR, de Villiers PJT, Kent AP. The Delphi technique in health sciences education research. Med Teach. 2005;27:639–43.

64. Niederberger M, Spranger J. Delphi Technique in Health Sciences: A Map. Front Public Health. 2020;0.

65. Hasson F, Keeney S, McKenna H. Research guidelines for the Delphi survey technique. Journal of Advanced Nursing. 2000;32:1008–15.

66. Eubank BH, Mohtadi NG, Lafave MR, Wiley JP, Bois AJ, Boorman RS, et al. Using the modified Delphi method to establish clinical consensus for the diagnosis and treatment of patients with rotator cuff pathology. BMC Medical Research Methodology. 2016;16:56.

67. Hsu C-C, Sandford B. The Delphi Technique: Making Sense Of Consensus. Practical Assessment, Research and Evaluation. 2007;12.

68. Bentley M, Kerr R, Powell S. The Use of a Modified Delphi Technique to Inform the Development of Best Practice in Interprofessional Training for Collaborative Primary Healthcare. Journal of Research in Interprofessional Practice and Education. 2016;6.

69. Woodcock T, Adeleke Y, Goeschel C, Pronovost P, Dixon-Woods M. A modified Delphi study to identify the features of high quality measurement plans for healthcare improvement projects. BMC Medical Research Methodology. 2020;20:8.

70. Koehn ML, Charles SC. A Delphi Study to Determine Leveling of the Interprofessional Core Competencies for Four Levels of Interprofessional Practice. MedSciEduc. 2019;29:389–98.

71. Veugelers R, Gaakeer MI, Patka P, Huijsman R. Improving design choices in Delphi studies in medicine: the case of an exemplary physician multi-round panel study with 100% response. BMC Medical Research Methodology. 2020;20:156.

72. Visser CLF, Ket JCF, Croiset G, Kusurkar RA. Perceptions of residents, medical and nursing students about Interprofessional education: a systematic review of the quantitative and qualitative literature. BMC Medical Education. 2017;17:77.

73. Gilligan C, Outram S, Levett-Jones T. Recommendations from recent graduates in medicine, nursing and pharmacy on improving interprofessional education in university programs: a qualitative study. BMC Medical Education. 2014;14:52.

74. Darlow B, Coleman K, McKinlay E, Donovan S, Beckingsale L, Gray B, et al. The positive impact of interprofessional education: a controlled trial to evaluate a programme for health professional students. BMC Medical Education. 2015;15:98.

75. Zechariah S, Ansa BE, Johnson SW, Gates AM, Leo GD. Interprofessional Education and Collaboration in Healthcare: An Exploratory Study of the Perspectives of Medical Students in the United States. Healthcare (Basel). 2019;7:117.

76. Jung H, Park KH, Min YH, Ji E. The effectiveness of interprofessional education programs for medical, nursing, and pharmacy students. Korean J Med Educ. 2020;32:131–42.

77. Topor DR, Dickey C, Stonestreet L, Wendt J, Woolley A, Budson A. Interprofessional Health Care Education at Academic Medical Centers: Using a SWOT Analysis to Develop and Implement Programming. MedEdPORTAL. 2018;14.

78. Fewster -Thuente Lori. A Contemporary Method to Teach Collaboration to Students. Journal of Nursing Education. 2014;53:641–5.

79. Zhang C, Thompson S, Miller C. A Review of Simulation-Based Interprofessional Education. Clinical Simulation In Nursing. 2011;7:e117–26.

80. Wilhelmsson M, Pelling S, Ludvigsson J, Hammar M, Dahlgren L-O, Faresjö T. Twenty years experiences of interprofessional education in Linköping – ground-breaking and sustainable. null. 2009;23:121–33.

81. Tran C, Kaila P, Salminen H. Conditions for interprofessional education for students in primary healthcare: a qualitative study. BMC Medical Education. 2018;18:122.

82. Buring SM, Bhushan A, Broeseker A, Conway S, Duncan-Hewitt W, Hansen L, et al. Interprofessional Education: Definitions, Student Competencies, and Guidelines for Implementation. Am J Pharm Educ. 2009;73:59.

83. Abu-Rish EL, Kim S, Choe L, Varpio L, Malik E, White AA, et al. Current Trends in Interprofessional Education of Health Sciences Students: A Literature Review. J Interprof Care. 2012;26:444–51.

84. Grymonpre RE. Faculty development in interprofessional education (IPE): Reflections from an IPE coordinator. Journal of Taibah University Medical Sciences. 2016;11:510–9.
