# Supplementary material for: Using the modified Delphi technique to develop a framework for interprofessional education during international electives in health professions training institutions in Sub-Saharan Africa
Source: Front Med (Lausanne). 2023 Oct 18;10:1225475. doi: 10.3389/fmed.2023.1225475 (PMC10618419; doi:10.3389/fmed.2023.1225475)
Supplement: Supplementary file 2 [file Data_Sheet_2.PDF]

Appendix 2: Round 1Delphi Technique Panel Questions

Consensus will be at ≥70% per question if the panel selected agree or strongly agree

Panel Questions	Agree (%)	Disagree (%)
**Relevance/ Suggestions of IPECP training in IEs**		
Interprofessional training needs to be integrated across international Electives placements in various health disciplines		
More evidence is needed on the organizational and systemic facilitators, determinants, and barriers of interprofessional education for collaborative practice in international electives		
IPE in International Electives will foster efficient multinational teamwork and IPC among different countries, especially in epidemics and pandemics		
It will lead to overall improved quality of health care at the patient, community, institution, and personal levels		
This framework will guide students, faculty, institutional leaders, and Administration on how to effectively structure and implement IPE electives in Health Training Institutions		
**Organizational /Operational needs**		
Committed home and host institutional leadership to supporting the program		
Effective and well-oriented administrative support to lead student's logistical needs and preparation		
Committed IPE faculty at the host institution to support student learning and training		
Effective agreements that allow reciprocity with home and host institutions		
Learning facilities, infrastructure, and premises to aid student learning		
Effective and clear application system in place to guide students on application requirements		
Effective communication between home and host institution during preparations		
Adequate financial support to cater to logistical costs		
Students from 2 or more different professional disciplines from home and host institutions (preferably those in the clinical training years)		
The IPE student groups during the elective placement at host institutions should include a minimum of 2 or more disciplines		
Each IPE student group during the elective should have 2-8 students to enable adequate learning		
**Acculturation needs**		

Effective Pre elective orientation courses/_workshops offered to students, faculty, clinical and community instructors, and administrators to enable effective understanding of roles, expectations, and the domains of IPEC training, culture, setting, and flow of activities		
Onsight Orientation by the admin and supervising faculty		
**Defining and understanding Competencies to be gained**<br>**This section covers knowledge, competencies, and capabilities for interprofessional education and training during IEs in any discipline of choice**<br>.		
Interprofessional learning outcomes relating to teamwork i.e Knowledge of, and skills for, teamwork		
Interprofessional learning outcomes related to roles and responsibilities i.e Knowledge and understanding of the different roles, boundaries, responsibilities, and expertise of health professionals		

Being able to challenge misconceptions in relation to roles		
Interprofessional learning outcomes related to communication i.e<br>Ability to communicate effectively with other health professional students		
Awareness of cultural differences in health profession command and conduct in another country		
Ability to express one's opinions with others involved in patient care		
Interprofessional learning outcomes relating to learning/reflection i.e Ability to reflect critically and evaluate their performance and that of the team		
Ability to transfer interprofessional learning gained during the international elective back home in the clinical, community, or public health setting		
Interprofessional learning outcomes relating to the patient/client i.e. Ability to recognize the central role of the patient in collaborative care		
Interprofessional learning outcomes relating to ethics/attitudes ie<br>Ability to acknowledge views and ideas of other professionals during an international elective placement		
Understanding the ethical issues relating to teamwork		
**IPEC Teaching Approaches that can be utilized during International Electives**		
Simulation-based IPE training		
Observership-based interprofessional learning		

Team-based approaches during clinical ward rounds and bedside teaching		
Community placements with the local students from various health disciplines		
Case study-based interprofessional learning with the local students		
Lecture/seminar-based education and training sessions		
**IPEC Learners Assessment Approaches during international Electives**		
Pre-post course knowledge/skills/attitude surveys		
Peer to peer Assessment		
Self-assessment/reflection (metacognitive skills) (Elective Report)		
Portfolio-based assessments (collection and review of individual and group work projects or assignments done)		
Team Observed Structured Clinical Examination (TOSCE)		
Simulated cases involving inter-professional practice		
Group feedback sessions		
**Mode of Elective Delivery**		
Online utilizing the teaching and assessment approaches that can be applied in a virtual platform eg country-specific case studies		
Actual outbound mobility physical mobility to a specific host institution		
Blended approach with both online and actual mobility		
**COVID 19 / Considerations**		
Adherence to the public health guidelines for home and host institutions		
The blended model can be used		
Negative COVID 19 tests before and after elective placement		
Vaccination is mandatory before rotation		
Wearing PPE and hand sanitization always		
Fewer cohorts of students hosted at a time		
Social distancing in all activities		

Adapted from (Bentley et al., 2016) and modified to suit this study
