# Supplementary material for: Using the modified Delphi technique to develop a framework for interprofessional education during international electives in health professions training institutions in Sub-Saharan Africa
Source: Front Med (Lausanne). 2023 Oct 18;10:1225475. doi: 10.3389/fmed.2023.1225475 (PMC10618419; doi:10.3389/fmed.2023.1225475)
Supplement: Supplementary file 3 [file Data_Sheet_3.PDF]

Appendix 3: Round 3 Delphi Guide Consensus

Consensus ≥70% with Agree responses

**Home and Host Training Institution's operational needs for IPE during International Electives**	**Agree**	**Disagree**
Home and host institutional leadership support for IPE in IEs programs		
Home and host institution administrative support to handle students' logistical needs before, during, and after the IE placement		
Faculty trained in IPE at the host institution to support and supervise students		
Partnership agreements that explore and allow reciprocity with home and host institutions		
Learning facilities to aid student learning		
Clear application system in place to guide students on IPE elective application requirements		
Communication strategy between home and host institution during preparations, implementation, and post participation		
Adequate financial support to cater to students' logistical costs		
Students from 2 or more different professional disciplines from home and host institutions (preferably those in the clinical training years)		
The IPE student groups during the elective placement at host institutions should include a minimum of 2 or more disciplines		
Each IPE student group during the elective should have 2-8 students to enable adequate learning		
**Acculturation Considerations**		
Pre-elective IPE orientation didactic sessions or seminars offered by the host institution to students, to enable understanding of roles, expectations, the domains of IPE, and the flow of activities		
Pre-Elective IPE training (workshops or seminars) offered to faculty, clinical and community instructors, to enable understanding of roles, expectations, the domains of IPE, and the flow of activities		
Onsite Orientation by the host institution on various social aspects and living to enable acclimatization of students in consideration of language, cultural humility, and equity.		

**Competencies to be gained by students participating in IPE international electives**<br>**By the end of the international elective students should be able to ;**		
Demonstrate Knowledge and attitudes, and skills for, teamwork		
Demonstrate knowledge and understanding of the different roles, boundaries, responsibilities, and expertise of various health professionals in the team		
communicate effectively and respectfully with other health professionals' students, faculty, patients, community, etc		
Demonstrate an awareness of cultural differences in health profession command and conduct in another country		
Express one's opinions with others involved in patient care with respect and humility		
Reflect critically and evaluate their performance and that of the team		
Develop a plan on how to apply interprofessional education and skills gained during the International elective back home in the clinical, community, or public health setting		
Recognize the central role of the patient/ community in collaborative care		
Acknowledge views and ideas of other professionals during an international elective		
**IPE teaching approaches that can be utilized during International Electives at Host institutions**		
Simulation-based IPE teaching		
Interprofessional community placements		
Country-Specific case study based interprofessional teaching		
joint tutorials using a flipped-classroom approach		
joint clinical placements through joint ward rounds and bedside teaching		
**IPE learner's Assessment Approaches during international electives at Host Institutions**		
**Formative (ongoing assessment)**		
Pre-elective course knowledge/skills/ Attitudes Surveys		
Portfolio-based assessments (collection and review of individual and group work projects or assignments done)		
Simulated cases involving interprofessional practice		
Peer to Peer assessment		

Team Objective Structured Clinical Examination (TOSCE)		
**Summative Assessment (End of Program Assessment)**		
Post Elective course knowledge/skills/attitude surveys		
Self-reflection through Elective Report at the end		
Team Objective Structured Clinical Examination (TOSCE)		
Simulated cases involving interprofessional practice		
Group feedback sessions		
**Mode of Elective Delivery**		
Online: utilizing the teaching and assessment approaches that can be applied in a virtual platform e.g. country-specific case studies		
Actual outbound physical mobility to a specific host institution		
Blended approach with both online and actual mobility at the host institution		
**Public Health Considerations**		
Adherence to the public health national guidelines for home and host institutions and countries with respect to health and safety requirements for traveling trainees.		
