# Supplementary material for: Using the modified Delphi technique to develop a framework for interprofessional education during international electives in health professions training institutions in Sub-Saharan Africa
Source: Front Med (Lausanne). 2023 Oct 18;10:1225475. doi: 10.3389/fmed.2023.1225475 (PMC10618419; doi:10.3389/fmed.2023.1225475)
Supplement: Supplementary file 4 [file Data_Sheet_4.DOCX]

**Appendix 4: Framework Guide for Implementation of Interprofessional Education (IPE) in International Electives in Health Professional Education Institutions**

**Introduction / Aim**

This framework aims to guide health education institutions on how to incorporate and implement interprofessional education (IPE) in international electives for health professionals and allied health programs. Our framework consists of 3 sections:

Section 1: Interprofessional Education and Collaborative Practice (IPECP) competencies to be gained

Section 2: Structuring of the IPECP- International Electives (IEs) programs.

Section 3: Implementation Guide for the delivery of IPECP-IEs at health professions education institutions

**Definition of terms**

**Inter-Professional Education (IPE)** refers to occasions when learners from two or more professions learn together; with, from, and about each other during all or part of their professional training with the objective of cultivating collaborative practice for providing client- or patient-centered health care [1].

**Interprofessional Collaborative Practice (ICP/IPC)** in health care occurs when multiple health workers from different professional backgrounds provide comprehensive services by working with patients, their families, careers, and communities to deliver the highest quality of care across settings [1].

**International Electives (IEs)** are defined as the time of learning where students have a choice on where to learn from, and what discipline they should be taking[2]. Students apply to open international electives at the host institutions in another country, undertaking learning in a specified selected discipline of choice. These occur in partnership with institutions that act as home and host institutions

**Acculturation**; is a process in which an individual adopts, acquires, and adjusts to a new cultural environment as a result of being placed into a new culture, or when another culture is brought to them [3].

**Multilateral Agreements:** This is where 2 or more institutions agree to partner with each institution owing to their equitable respective obligations to all the other parties/institutions involved [4].

**Home Institution:** is the institution at which the exchange student is registered as a full-time student and sends the student to another ( Host) Institution [5]. The Home institution is the sending institution.

**Host Institution:** is an institution that receives or accepts students from another institution in a well-defined partnership to pursue training that could be short or long-term [6]. The host institution is the receiving institution.

**Flipped Classroom:** This is a type of blended learning which aims to increase student engagement and learning by having students complete readings and assignments outside of class and have a discussion on the readings and assignments during the classroom session with the active participation of all students [7]

**Team Objective Structured Clinical Examination (TOSCE):** This is a type of assessment where students move to various stations as a team with varying clinical cases and situations to evaluate their performance as a team [8]. This is mainly done in the clinical area

**Community Placement:** This is the time of learning where students spend time learning about and experiencing the health of the population in their neighborhoods outside of the clinical setting [9].

**Online Learning Approach:** this is an educational approach that utilizes digital aspects such as web-based platforms and the internet to deliver knowledge and skills among learners [10].

**Blended Learning Approach:** this is an educational approach that utilizes both the online learning approach and the in-person classroom approach to deliver knowledge and skills among learners [11].

**Section 1: Interprofessional Education and Collaborative Practice (IPECP) Competencies to be gained**

| **Competencies to be gained by students participating in IPECP- IEs.**  **By the end of the international elective students should be able to ;** |
| --- |
| Demonstrate Knowledge and attitudes, and skills for, teamwork |
| Demonstrate knowledge and understanding of the different roles, boundaries, responsibilities, and expertise of various health professionals in the team |
| communicate effectively and respectfully with other health professionals’ students, faculty, patients, community, etc |
| Demonstrate an awareness of cultural differences in health profession command and conduct in another country |
| Express one's opinions with others involved in patient care with respect and humility |
| Reflect critically and evaluate their performance and that of the team |
| Develop a plan on how to apply interprofessional education and skills gained during the international elective back home in the clinical, community, or public health setting |
| Recognize the central role of the patient/ community in collaborative care |
| Acknowledge views and ideas of other professionals during an international elective |

**Section 2; Structuring of the IPECP-IEs Programs**

| **Home and Host Training Institution's operational needs for IPE during International Electives** |
| --- |
| Home and host institutional leadership support for IPECP in IEs programs |
| Home and host institution administrative support to handle students’ logistical needs before, during, and after the IE placement |
| Faculty trained in IPECP at the host institution to support and supervise students |
| Partnership agreements that explore and allow reciprocity with home and host institutions |
| Learning facilities to aid student learning |
| Clear application system in place to guide students on IPE elective application requirements |
| Communication strategy between home and host institution during preparations, implementation, and post participation |
| Adequate financial support to cater to students’ logistical costs |
| Students from 2 or more different professional disciplines from home and host institutions (preferably those in the clinical training years) |
| The IPECP student groups during the elective placement at host institutions should include a minimum of 2 or more disciplines |
| Each IPECP student group during the elective should have 2-8 students to enable adequate learning |
| **Acculturation Considerations** |
| Pre-elective IPECP orientation didactic sessions or seminars offered by the host institution to students, to enable understanding of roles, expectations, the domains of IPE, and the flow of activities |
| Pre-Elective IPECP training (workshops or seminars) is offered to both home and host institution faculty, and clinical and community instructors, to enable understanding of roles, expectations, the domains of IPE, and the flow of activities at home and host institutions. |
| Onsite Orientation by the host institution on various social aspects and living to enable acclimatization of students in consideration of language, cultural humility, and equity. |
| **IPECP teaching approaches that can be utilized during International Electives at Host institutions** |
| Simulation-based IPECP teaching |
| Interprofessional community placements |
| Country-Specific case study based interprofessional teaching |
| joint tutorials using a flipped-classroom approach |
| joint clinical placements through joint ward rounds and bedside teaching |
| **IPECP learner's Assessment Approaches during international electives at Host Institutions** |
| **Formative ( ongoing assessment )** |
| Pre-elective course knowledge/skills/ Attitudes Surveys |
| Portfolio-based assessments ( collection and review of individual and group work projects or assignments done) |
| Simulated cases involving interprofessional practice |
| Peer to Peer assessment |
| Team Objective Structured Clinical Examination (TOSCE) |
| **Summative Assessment ( End of Program Assessment)** |
| Post Elective course knowledge/skills/attitude surveys |
| Self-reflection through Elective Report at the end |
| Team Objective Structured Clinical Examination (TOSCE) |
| Simulated cases involving interprofessional practice |
| Group feedback sessions |
| **Mode of Elective Delivery** |
| Online: utilizing the teaching and assessment approaches that can be applied in a virtual platform e.g., country-specific case studies |
| Actual outbound physical mobility to a specific host institution |
| Blended approach with both online and actual mobility at the host institution |
| **Public Health Considerations** |
| Adherence to the public health national guidelines for home and host institutions and countries with respect to health and safety requirements for traveling trainees. |

**Section 3: Implementation Guide for the delivery of IPECP during International Electives (IEs) in Health Professions Education Institutions**

This guide is developed following the framework developed in sections 1 and 2. In bold are the various constructs of the framework and below each are points that could be considered during the implementation of IPECP during IEs. Next is a check box that can allow you to check off as you use the guide to guide your implementation

| **Home and Host Training Institution's operational needs for IPECP during International Electives** | **Tick off what has been used in each of the sections to guide your IPECP -IEs** |
| --- | --- |
| **Home and host institutional leadership support for IPECP in IEs programs** |  |
| Both institutions' leadership submission of their expression of interest to each other after an IPECP-IEs program overview. |  |
| Endorse the IPECP-IEs program, Sign MOUs/Agreements, and lead the program to the official institutional structure to support the program i.e., admin, faculty selection, etc. |  |
| Respond to emails |  |
| **Home and host institution administrative support to handle students and faculty logistical needs before, during, and after the IE placement** |  |
| Have a program manager or international office handle this at both home and host institution  Assigned handling person at home and host institution to have an IPECP-IEs orientation overview meeting to enable generic understanding of the program  IPECP-IEs Program manager to   - Review applications, consult with host faculty for application review, and then proceed with acceptance notice. - Provide guidelines on the student preparation checklist. (If blended and physical mobility, assist with providing guidance on visa applications, Institutional requirements, ground transport and airport pick up, etc) - If the program is virtual, provide an overview of the program aims, teaching plan, learning materials, platform to be used for learning, etc - Provide an acculturation and orientation session to the students - Develop a clear compensation plan for the faculty time and logistical support to students - Manage all financial implications with proper accountability and documentation - All other roles that may arise |  |
| **Faculty trained in IPECP at the host institution to support and supervise students** |  |
| At least two faculty from different health professions e.g., pharmacy, medicine, nursing, Dentistry, etc. The selected faculty should have:   - Interest and involvement in IPECP - Experience in supervising and supporting international students - community placements and field supervision, - Prior IPECP teaching experience is an added advantage |  |
| **Partnership agreements that explore and allow reciprocity with home and host institutions** |  |
| Have a unifying agreement that binds all schools participating in the IPECP-IEs program with well-defined roles for both home and host institutions |  |
| **Learning facilities to aid student learning** |  |
| **Physical Interaction:** Teaching hospitals, lecture rooms, simulation/ skills labs, elective curriculum, teaching plans, and teaching or learning materials for students to use |  |
| **Virtual Interaction:** Internet connection, online learning platforms, elective curriculum, teaching plans, teaching, and learning materials |  |
| **Clear application system in place to guide students on IPECP elective application requirements** |  |
| Application system that gives a detailed overview of the electives available with the ability to allow students to upload supporting documents irrespective of their country location |  |
| A clear submission guide with the ability to identify when electives will be open |  |
| Extra requirements specific to IPECP, E.g., if IPECP-IEs, requiring students to apply as a team of 2 or more students from 2 or more disciplines |  |
| **Communication strategy between home and host institution during preparations, implementation, and post participation** |  |
| Regular email communications |  |
| **Adequate financial support to cater to students and Faculty logistical costs** |  |
| Funds to compensate students' needs i.e. if the online mode of delivery is used then funds to subscribe for stable internet. |  |
| If blended and physical mobility, funds to cater for tickets, insurance, vaccination, professional registration or licensure, accommodation, and meals. This can be from the institutions, a partner organization, or self-funding by the students |  |
| **Students from 2 or more health professions from home and host institutions (preferably those in the clinical training years)** |  |
| These can be from Medicine, Nursing, Pharmacy, Dentistry, Physiotherapy, lab medicine, Biomedical Sciences, radiology, etc. |  |
| **The IPECP student groups during the elective placement at host institutions should include a minimum of two health professions** |  |
| This will depend on cohort size |  |
| **Each IPECP elective student group should have 2-8 students to enable adequate learning** |  |
| A cohort of 5 students per institution is an example but grouping to what best works for each institution is recommended. |  |
| **Acculturation Considerations** |  |
| **Pre-elective IPECP orientation didactic sessions or seminars offered by the host institution to students, to enable understanding of roles, expectations, the domains of IPECP, and the flow of activities** |  |
| In-person live sessions, Synchronous or asynchronous sessions e.g., Voice over PowerPoint, recorded lecture, and in-person lecture sessions that give:   - An overview of the program, - Aims of the program, - Define IPE and IPC, - Define students' roles and expectations as they participate, - The flow of activities, - Duration of elective |  |
| **Pre-Elective IPECP training (workshops or seminars) is offered to both home and host institution faculty, and clinical and community instructors, to enable understanding of roles, expectations, the domains of IPE, and the flow of activities at home and host institutions** |  |
| Conduct an online/in-person workshop delivered by IPECP experts to enable them to understand:  The IPECP-IEs program (background, overview, aims, span, etc.)   - - The structure, acculturation, and principles for student participation   - Faculty roles   - IPECP definitions and core competencies   - The importance of IPECP in health care   - The various teaching methods for IPECP virtually   - The various learning assessment methods for IPECP virtually |  |
| **Onsite Orientation by the host institution on various social aspects and living to enable acclimatization of students in consideration of language, cultural humility, and equity.** |  |
| **If physical mobility**; this can be done by the international office, student community at the home institution, and faculty in the first week of arrival specifically on day 1. This can then be followed with continuous interaction in the first week and throughout the elective. This can have topics on host country overview in general, language, school campus overview, way of life, food availability, transport means, accommodation, weather, currency, dress code, places to get needs, institutional culture, and the population that will be interacted with, orientation to the department, understanding the workflows and departments culture, safety precautions, etc.  Incoming Students (students taking the elective in another institution) should also be able to share their culture and home reality so that there is dual learning from both home and host students and faculty interactions |  |
| **If online;** the first session with the faculty and program manager will focus on this at the home institution, continued online interaction, and social communication groups throughout the program**.**  Both the students and faculty should be able to give an overview of their countries' culture, school culture, language preferences, way of life, etc. |  |
| **IPECP teaching approaches that can be utilized during International Electives at Host institutions** |  |
| **Simulation-based IPECP teaching**   - Utilize the simulation centers and skills labs if available for students with an aim of IP Teams Engagement |  |
| **Interprofessional community placements**   - Utilize the existing structure of local students’ community placements and place the IE students in one of the sites with effective supervision where the local students go |  |
| **Country-Specific case study based interprofessional teaching**   - These should extensively describe the issue at hand and have some probe questions to stimulate student joint engagement, through understanding the issue and innovatively addressing the issue at hand as a team |  |
| **Joint tutorials using a flipped-classroom approach**   - Have sessions where students have a topic to read about, work on as a team or through Self Directed Learning and during the joint session have a discussion and activities on what was learned and applied |  |
| **Joint clinical placements through joint ward rounds and bedside teaching**   - This will depend on the discipline of choice, but it will include the interprofessional cohort of students joining the ward round and bedside teaching and conducting learning from, with, and about each other with a patient-centered approach |  |
| **IPECP learner's Assessment Approaches during international electives at Host Institutions** |  |
| **Formative ( ongoing assessment )** |  |
| **Pre-elective course knowledge/skills/ Attitudes Surveys**   - Could be based on already existing validated IPECP skills attainment scales/ tools and can be built online using an online survey platform or given as hard copies. Examples among others include the - The 2018 Revised Interprofessional Collaborative Competency Attainment Scale (ICCAS 2018) [12] |  |
| **Portfolio-based assessments**   - collection and review of individual and group work projects or assignments done |  |
| **Simulated cases involving interprofessional practice**   - Utilize the simulation centers and skills labs if available for students to assess IP Teams Engagement and application of IPECP skills |  |
| **Peer to Peer assessment**   - This can be done during a joint session that is synchronous if online or in-person if blended or physical mobility |  |
| **Team Observed Structured Clinical Examination (TOSCE)**   - Design stations where students go to these stations in teams and perform the instruction at hand as the faculty assess and gives feedback on how they apply teamwork and IPC skills |  |
| **Summative Assessment ( End of Program Assessment)** |  |
| **Post Elective course knowledge/skills/attitude surveys**   - Could be based on already existing validated IPECP skills attainment scales/ tools and can be built online using an online survey platform or given as hard copies. Examples among others include the - The 2018 Revised Interprofessional Collaborative Competency Attainment Scale (ICCAS 2018) [12] - EIPEN Behavioural indicators of key competences for interprofessional practice assessment tool [13] |  |
| **Self-reflection through Elective Report at the end**   - A uniform template should be given to enable students to make a well-structured report in all domains of the entire rotation including IPECP skills gained |  |
| **Team Observed Structured Clinical Examination (TOSCE)**   - Design stations where students go to these stations in teams and perform the instruction at hand as the faculty assess and gives feedback on how they apply teamwork and IPC skills |  |
| **Simulated cases involving interprofessional practice**  Utilize the simulation centers and skills labs if available for students to assess IP Teams Engagement and application of IPECP skills |  |
| **Group feedback sessions**   - Can be done online or in person by having students jointly report on the IPECP gained, their experiences, and main takeaways with the aid of PowerPoint presentations |  |
| **Mode of Elective Delivery** |  |
| **Online: utilizing the teaching and assessment approaches that can be applied in a virtual platform e.g., country-specific case studies** |  |
| This can be done online using zoom, an LMS system, with clear identification of the teaching plan, with both synchronous and asynchronous activities and clear meeting times for synchronous sessions  Synchronous sessions with the faculty and students should happen at a minimum of once a week and the duration should be as agreed by the faculty and students e.g., 1-1.5hours per session.  Duration 6-8 weeks |  |
| **Actual outbound physical mobility to a specific host institution** |  |
| This involves attachment to a specific department and thus having to do joint clinical ward rounds, bedside teaching, and all activities in a particular rotation with emphasis on IPECP objectives.    Duration 6-8 weeks |  |
| **Blended approach with both online and actual mobility at the host institution** |  |
| This has both the online and actual mobility components, the duration for physical placements should be more than the online.  E.g., for a 6-week rotation, online interaction could be 2 weeks and then 4weeks for physical mobility |  |
| **Public Health Considerations** |  |
| Adherence to the public health national guidelines for home and host institutions and countries with respect to health and safety requirements for traveling trainees.   - Paying attention to vaccinations required, Medical Tests required, health insurance, Personal Protection Equipment, and attention to personal safety guidelines given by the school if it is done by blended or physical mobility approach |  |
|  |  |

**References**

1. World Health Organization. Framework for action on interprofessional education & collaborative practice. 2010. https://www.who.int/publications-detail-redirect/framework-for-action-on-interprofessional-education-collaborative-practice. Accessed 25 Apr 2022.

2. Grudzen CR, Legome E. Loss of international medical experiences: knowledge, attitudes and skills at risk. BMC Med Educ. 2007;7:47.

3. Rudmin F, Wang bo, Castro J. Acculturation Research Critiques and Alternative Research Designs. In: The Oxford Handbook of Acculturation and Health. 2016. p. 25.

4. Aust A. Modern Treaty Law and Practice. Higher Education from Cambridge University Press. 2013. https://www.cambridge.org/highereducation/books/modern-treaty-law-and-practice/94B8FEA58D2989D9267557F6118D4B35. Accessed 25 Apr 2022.

5. Law Insider. Home Institution Definition: 158 Samples. Law Insider. 2022. https://www.lawinsider.com/dictionary/home-institution. Accessed 25 Apr 2022.

6. Law Insider. Host Institution Definition: 205 Samples. Law Insider. 2022. https://www.lawinsider.com/dictionary/host-institution. Accessed 25 Apr 2022.

7. Ozdamli F, Asiksoy G. Flipped Classroom Approach. World Journal on Educational Technology: Current Issues. 2016;8:98–105.

8. Amini M, Moghadami M, Kojuri J, Abbasi H, Abadi AAD, Molaee NA, et al. Using TOSCE (Team Objective Structured Clinical Examination) in the second national medical sciences olympiad in Iran. J Res Med Sci. 2012;17:975–8.

9. Temple J. What constitutes a community placement? Nursing Times. 2013. https://www.nursingtimes.net/roles/nurse-educators/what-constitutes-a-community-placement-13-09-2013/. Accessed 28 Sep 2022.

10. Reese SA. Online learning environments in higher education: Connectivism vs. dissociation. Educ Inf Technol. 2015;20:579–88.

11. Chen AK, Dennehy C, Fitzsimmons A, Hyde S, Lee K, Rivera J, et al. Teaching interprofessional collaborative care skills using a blended learning approach. Journal of Interprofessional Education & Practice. 2017;8:86–90.

12. MacDonald CJ, Trumpower D, Archibald D. Interprofessional Collaborative Competencies Attainment Survey (ICCAS) Revised. National Center for Interprofessional Practice and Education. 2018. https://nexusipe.org/advancing/assessment-evaluation/interprofessional-collaborative-competencies-attainment-survey-iccas. Accessed 13 Mar 2023.

13. European Interproffesional Practice and Education Network. EIPEN Assessment sheet. Behavioural indicators of key competences for interprofessional practice. 2021.
